# Acute Optogenetic Stimulation of Serotonin Neurons Reduces Cell Proliferation in the Dentate Gyrus of Mice

**DOI:** 10.1021/acschemneuro.4c00771

**Published:** 2025-02-12

**Authors:** Naozumi Araragi, Markus Petermann, Mototaka Suzuki, Matthew Larkum, Valentina Mosienko, Michael Bader, Natalia Alenina, Friederike Klempin

**Affiliations:** † Max Delbrück Center for Molecular Medicine in the Helmholtz Association, Robert-Rössle-Straße 10, 13125 Berlin, Germany; ‡ Charité University Medicine Berlin, Charitéplatz 1, 10117 Berlin, Germany; § Department of Cognitive and Systems Neuroscience, Swammerdam Institute for Life Sciences, Faculty of Science, University of Amsterdam, 1098XH Amsterdam, The Netherlands; ∥ NeuroCure Cluster of Excellence, Department of Biology, Humboldt University, Charitéplatz 1, 10117 Berlin, Germany; ⊥ German Center for Cardiovascular Research (DZHK), Partner Site, 10785 Berlin, Germany; # Institute for Biology, University of Lübeck, 23562 Lübeck, Germany

**Keywords:** Optogenetics, ChR2, Tph2, dentate gyrus, BrdU

## Abstract

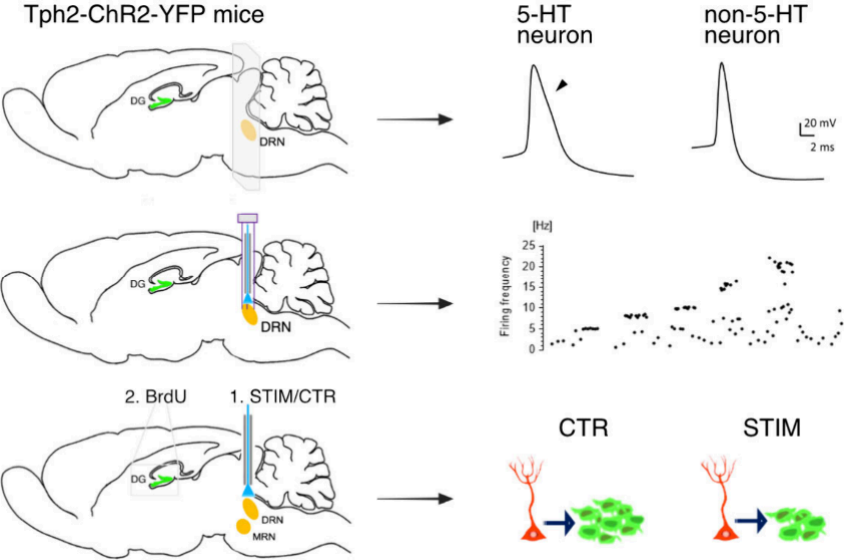

The dentate gyrus of the hippocampus is targeted by axons from serotonin raphe neurons, where the neurotransmitter modulates adult neurogenesis and antidepressant action, and mediates the neurogenic effect of running. Whether running-induced cell proliferation is directly mediated by serotonin remains unknown. Here, we took advantage of Tph2-ChR2-YFP transgenic mice in which the light-sensitive protein channelrhodopsin-2 (ChR2) is specifically expressed in tryptophan hydroxylase 2 (TPH2)-expressing neurons. We selectively activated serotonin neurons via optogenetics and determined the effect on cell proliferation in the dentate gyrus. Our data reveal a significant reduction in the number of newly generated cells upon overnight raphe stimulation. The decrease in cell proliferation was absent when serotonin neurons were light-activated for six consecutive nights. However, we observed an interhemispheric difference in BrdU-positive cell numbers. We conclude that acute network dynamics occur between serotonin raphe neurons and the hippocampus, directly affecting precursor cell proliferation.

## Introduction

Modulating both proliferation and survival of newly generated cells, serotonin (5-hydroxytryptamine, 5-HT) emerges as a key regulator of adult neurogenesis in the dentate gyrus of the hippocampus.^1^ Serotonin neurons are located in the brainstem’s raphe nuclei.^2,3^ Fiber projections originating from the median and dorsal raphe nuclei (MRN, DRN) terminate prominently in the hippocampus,^4,5^ where they synapse on principal neurons and interneurons. Target areas in the dentate gyrus express various 5-HT receptors that control the response from efferent activity.^6,7^ Hilar interneurons in the dentate gyrus appear to be preferentially innervated by 5-HT fibers from the MRN,^8,9^ suggesting an indirect effect of 5-HT action on immature and mature granule cells, with glutamate as the most prevalent cotransmitter.^10,11^ In the absence of brain 5-HT, hyperinnervation of raphe projections in the hippocampus is observed;^12^ nevertheless, the number of proliferating cells remains unchanged.^13^

Tryptophan hydroxylase-2 (TPH2) is the rate-limiting enzyme for 5-HT synthesis in the brain^14^ and is expressed exclusively in 5-HT neurons. Expression of Channelrhodopsin-2 (ChR2) fused to enhanced yellow fluorescent protein (mhChR2::eYFP) under control of the *Tph2* promoter in Tph2-ChR2-YFP transgenic mice allows for specific optogenetic activation and visualization of the 5-HT neurons.^15,16^ Our earlier data established 5-HT as the specific modulator of exercise-induced cell proliferation.^13,17^ However, whether running-induced 5-HT stimulation and neuronal plasticity are directly coupled remains unknown. Here, we utilized Tph2-ChR2-YFP mice together with optogenetics to determine whether activation of 5-HT raphe neurons directly affects cell proliferation in the hippocampus.

We characterized 5-HT and non-5-HT raphe neurons, including their cell morphology and physiological properties, in acute brain slices of Tph2-ChR2-YFP mice, and specifically examined dorsal raphe neurons in anesthetized mice using an optetrode. We determined the frequency and duration of light pulses required to activate 5-HT neurons in vivo to define downstream regulatory networks. Specifically, we investigated cell proliferation following an overnight stimulation of neurons in the DRN and MRN, and we assessed the effects of daily optogenetic stimulation of the DRN for 1 week, a time-period corresponding to the established running protocol.^13^

## Results and Discussion

### Properties of Tph2-ChR2-YFP Expressing Neurons in Acute Brain Slices

**Figure 1:**
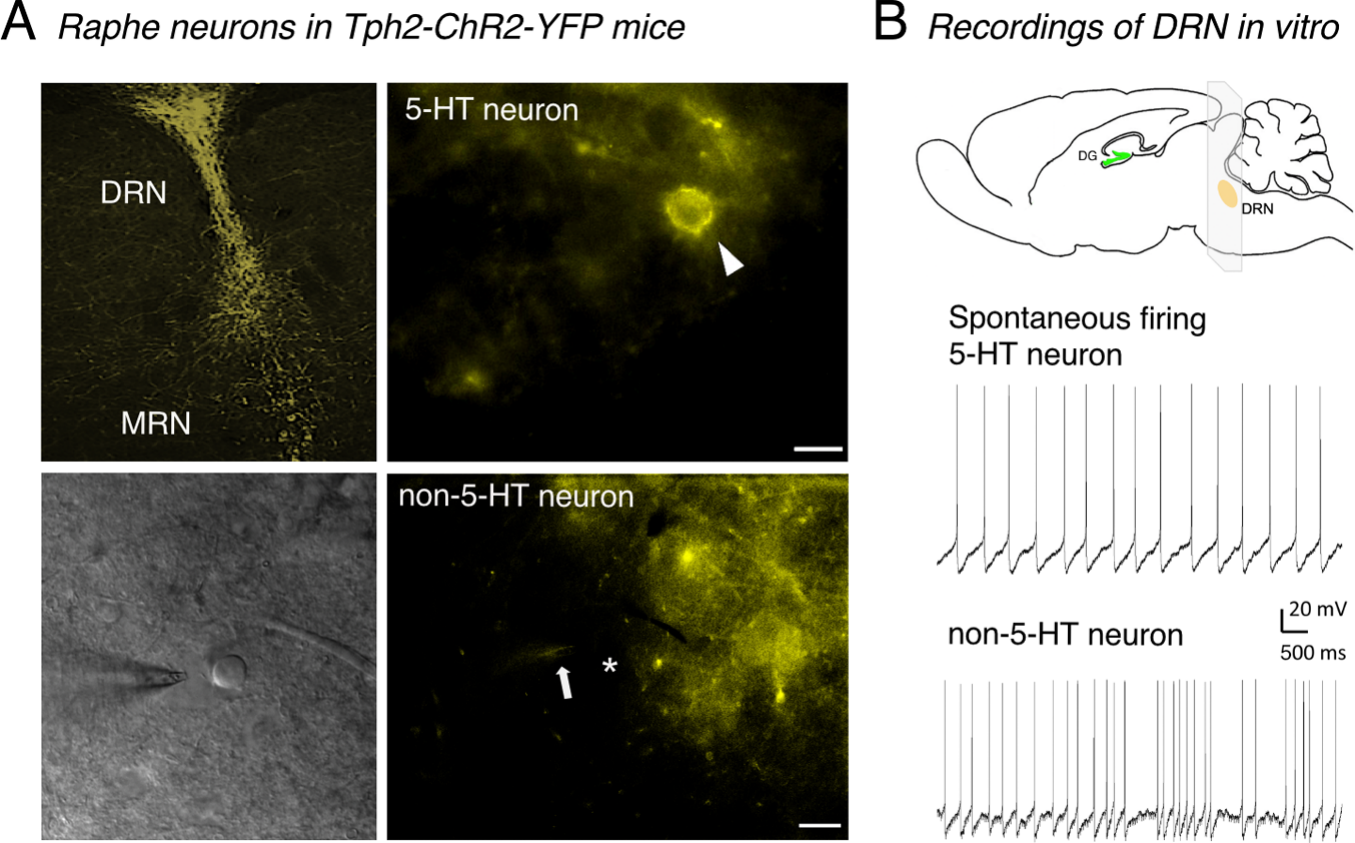
Comparison of 5-HT vs non-5-HT neurons in young-adult mouse brain slices. (A) Fluorescence images show Tph2-ChR2-YFP-positive neurons in brain stem dorsal (DRN) and median (MRN) raphe nuclei (top; arrowhead indicates 5-HT neuron), and a bright-field image displays a DRN slice indicating the position of the patch pipet during recording of a non-5-HT neuron (bottom, asterisk indicates the neuron; arrow indicates patch pipet). Scale bars represent 20 μm. (B) Acute slices of 200 μm were cut from the young-adult mouse brain to obtain the DRN, and spontaneous firing of 5-HT and non-5-HT neurons was recorded by patch-clamp. While 5-HT neurons exhibit a slow, tonic firing pattern, non-5-HT neurons fire rapidly and irregularly.

Serotonin neurons in the brainstem’s raphe nuclei were morphologically distinguished by their large cell bodies (∼20–25 μm), as has been described earlier,^18^ and YFP fluorescence. Previous studies have shown selective YFP expression in TPH2-positive cells in both DRN and MRN in Tph2-ChR2-YFP mice, confirming 5-HT identity of YFP-positive neurons.^15^ We observed strong YFP expression in somata and fibers in the DRN with moderate expression in the MRN (Figure 1A). Non-5-HT neurons in the DRN typically have relatively small somata (∼10–15 μm; Figure 1A). To determine the electrophysiological properties of neurons in the DRN, we recorded the spontaneous firing of 5-HT and non-5-HT neurons. While 5-HT neurons displayed a slow, rhythmic firing pattern (*n* = 8), non-5-HT neurons exhibited fast and irregular firing rates (*n* = 11; Figure 1B). Non-5-HT neurons include dopamine- and norepinephrine-containing cells, as well as glutamate-containing cells, which are sparsely located in the DRN. In addition, GABAergic cells are densely distributed.^19^

**Figure 2:**
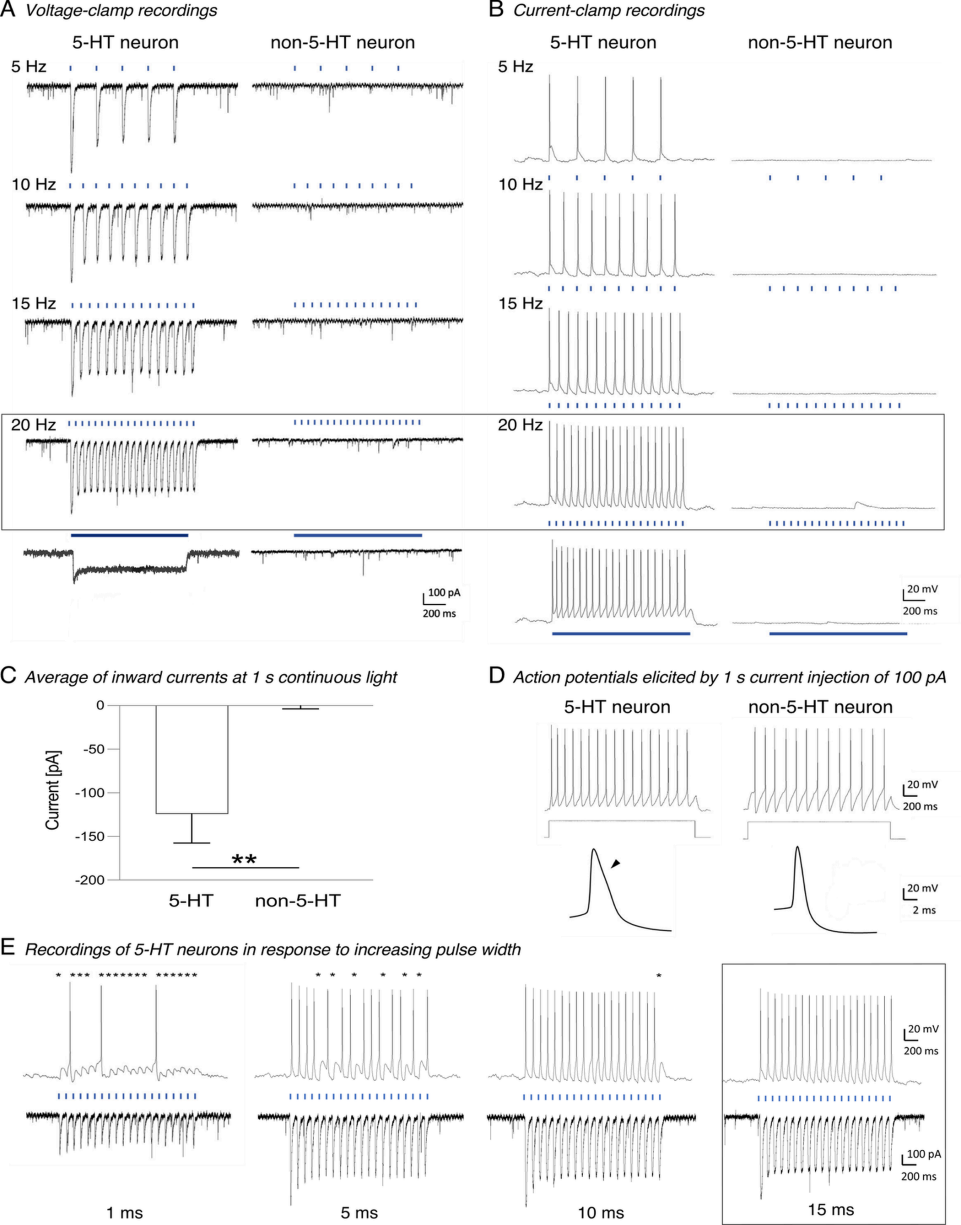
Physiological properties of Tph2-ChR2-YFP-expressing cells (5-HT neurons) in the dorsal raphe nuclei (DRN), compared to non-5-HT raphe neurons in young-adult mouse brain slices. (A, B) Recordings of inward currents were conducted by the voltage clamp (panel (A)), and firing activity of action potentials in current-clamp mode (panel (B)). Membrane currents were induced by LED light pulses lasting 15 ms (illustrated as blue ticks), and neurons were stimulated with increasing frequency from 5 to 20 Hz or with continuous light lasting 1 s (panels (A) and (B), bottom). The frequency of 20 Hz was selected for in vivo studies. (C) Average of inward currents in response to continuous light lasting 1 s ((**) *p* < 0.01). (D) Recordings in current-clamp mode, in response to 100 pA current injection. The average action potential shape (excluding the first few spikes) of a 5-HT neuron is wider (HHW = 2.7 ms), compared to a non-5-HT neuron (narrow, HHW = 1.5 ms); a shoulder in the repolarizing phase is indicated by an arrowhead. HHW refers to the half-height-width of an action potential. (E) Recordings in current-clamp (top) and voltage-clamp (bottom) modes in response to optogenetic stimulation with increasing pulse widths from 1 to 15 ms. A shorter pulse is more likely to fail to elicit an action potential (asterisks indicate such missed action potentials). A pulse duration of 15 ms was chosen for in vivo studies. Data are presented as mean + SEM.

Recordings of inward currents by voltage-clamp (Figure 2A) and the firing activity of action potentials elicited by the current-clamp technique (Figure 2B) are shown in Figure 2. Inward currents were evoked by applying light pulses of 15 ms, and neurons were stimulated with increasing frequencies from 5 to 20 Hz, or with 1 s continuous light. Firing activity of action potentials showed that at both 20 Hz and 1 s continuous light pulse, 5-HT neurons generated 20–21 action potentials (Figure 2B). We chose 20 Hz for in vivo studies since a 5-HT neuron can reliably elicit action potentials at ∼20 Hz. When quantifying inward currents in response to 1 s continuous light stimulation, 5-HT neurons showed large amplitudes (5-HT vs non-5-HT, −124.1 ± 33.46 pA vs −0.23 ± 3.50 pA, *p* = 0.0041, *n* = 8 and 11 cells, respectively Figure 2C), further confirming the cells’ identity.

The general physiology, shape, and duration of action potentials differ between 5-HT and non-5-HT neurons.^20,21^ As shown in Figure 2D, non-5-HT neurons in the DRN exhibit relatively brief and narrow action potentials due to faster repolarization (action potentials were elicited by 100 pA currents). In contrast, 5-HT neurons are characterized by broader, prolonged action potentials with a “shoulder” phase, which extends their duration (Figure 2D). Additionally, 5-HT and non-5-HT neurons differ in half-height width (HHW), amplitude, firing rate, and up-to-down stroke ratio. For 5-HT vs non-5HT neurons, the average action potential amplitude is 82.6 ± 3.87 mV vs 73.34 ± 7.06 mV, HHW is 2.47 ± 0.20 ms vs 1.39 ± 0.18 ms, firing rate is 10.33 ± 2.68 Hz vs 20.08 ± 6.22 Hz, and the up-to-down stroke ratio is 5.57 ± 0.52 vs 3.61 ± 0.27. Figure 2D illustrates a sample measurement, showing an HHW of 2.7 ms for a 5-HT neuron, compared to 1.5 ms for a non-5-HT neuron, as well as an asymmetric vs symmetric up-to-down stroke ratio.

We varied duration and intensity of the light stimulation. In response to optogenetic activation with increasing pulse widths from 1 to 15 ms, current-clamp recordings revealed that a shorter pulse is more likely to fail to elicit an action potential. Serotonin neurons started to respond to every light pulse at 15 ms (Figure 2E). Overall, our systematic approach of utilizing increasing frequencies and pulse widths revealed values of 20 Hz and 15 ms as the most suitable to effectively stimulate 5-HT neurons in Tph2-ChR2-YFP mice. We chose those values for the following in vivo studies.

### Firing Characteristics and Response Pattern of Tph2-ChR2-YFP Neurons In Vivo

**Figure 3:**
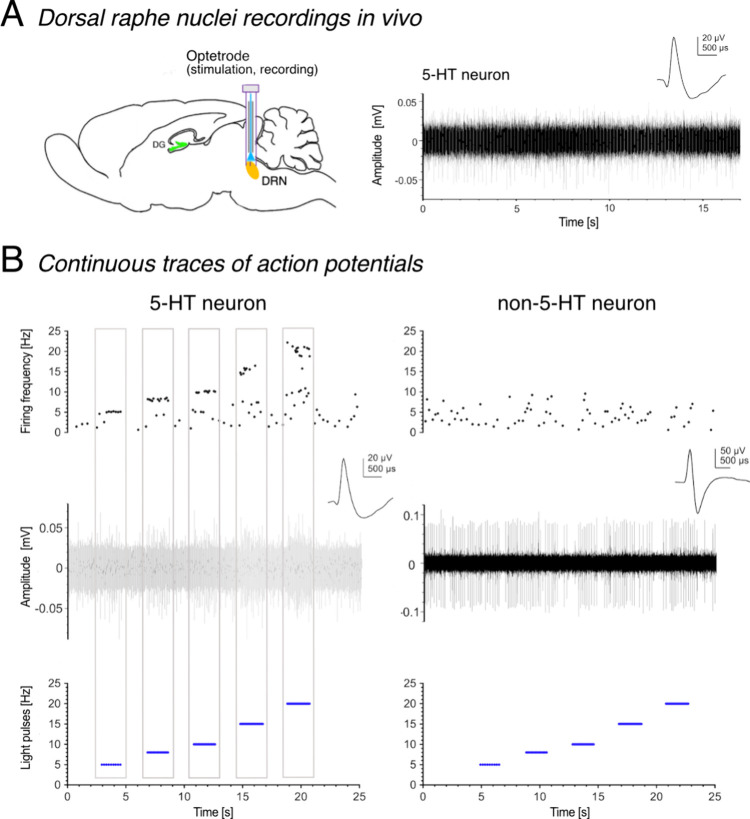
Firing characteristics and response pattern to light-stimulation of dorsal raphe neurons in vivo. (A) Tph2-ChR2-YFP-expressing cells in the dorsal raphe nuclei (DRN) were light-stimulated, and extracellular field potentials were subsequently recorded via an optetrode in anesthetized mice. The right panel displays a recorded continuous trace of a baseline-firing 5-HT neuron. (B) Continuous traces of action potentials recorded from a 5-HT and non-5-HT neuron. Upon stepwise increases in light frequencies (highlighted in blue, bottom), a temporally precise activation of 5-HT neurons was observed (left panel). At stimulation frequencies higher than 15 Hz, 5-HT neurons failed to reliably respond to an individual stimulus as characterized by single drops (top of left panel). Non-5-HT neurons did not respond to light stimulation (right panel).

We proceeded to in vivo studies using an optetrode enabling light stimulation and subsequent extracellular recordings of Tph2-ChR-YFP-expressing cells in the DRN of anesthetized mice (*n* = 4, Figure 3A). Figure 3B depicts the firing rates of 5-HT and non-5-HT neurons upon optogenetic activation with a stepwise increase in light frequencies (5–20 Hz). This light stimulation resulted in a temporally precise activation of action potentials and a progressive increase in their firing frequency. However, at stimulation frequencies higher than 15 Hz, 5-HT neurons did not always exhibit a one-to-one response to individual light stimuli, leading to occasional drops in firing frequency (Figure 3B, left panel). Notably, the shape of action potentials detected during light stimulation was identical to those detected at baseline (Figure 3A, right panel), thus excluding the possibility of optically induced electrical artifacts. Non-5-HT neurons did not respond to light stimulation (Figure 3B, right panel). Similar results were obtained from recordings in the MRN (data not shown). These data confirm that 5-HT neurons in the raphe nuclei of Tph2-ChR-YFP mice can be specifically activated by blue light.

Light spreads in a cone from the fiber tip, decreasing in intensity with tissue depth due to scattering and absorption. Effective neuronal activation requires sufficient light delivery, which can be achieved through an angled fiber implantation and LEDs. In our study, we used short light pulses (15 ms, 10–12 mW, 465 nm) delivered via a 200 μm optetrode to prevent tissue damage and achieve ∼1 mm spatial resolution, with effects spreading further through neuronal coupling. Importantly, in the DRN, volume transmission plays a key role, allowing 5-HT to diffuse through the extracellular space and enabling widespread neuromodulation. These findings emphasize the utility of precise optical techniques for controlling and studying the 5-HT network.

### Acute Stimulation of Tph2-ChR2-YFP Neurons Affects Cell Proliferation in the Dentate Gyrus

**Figure 4:**
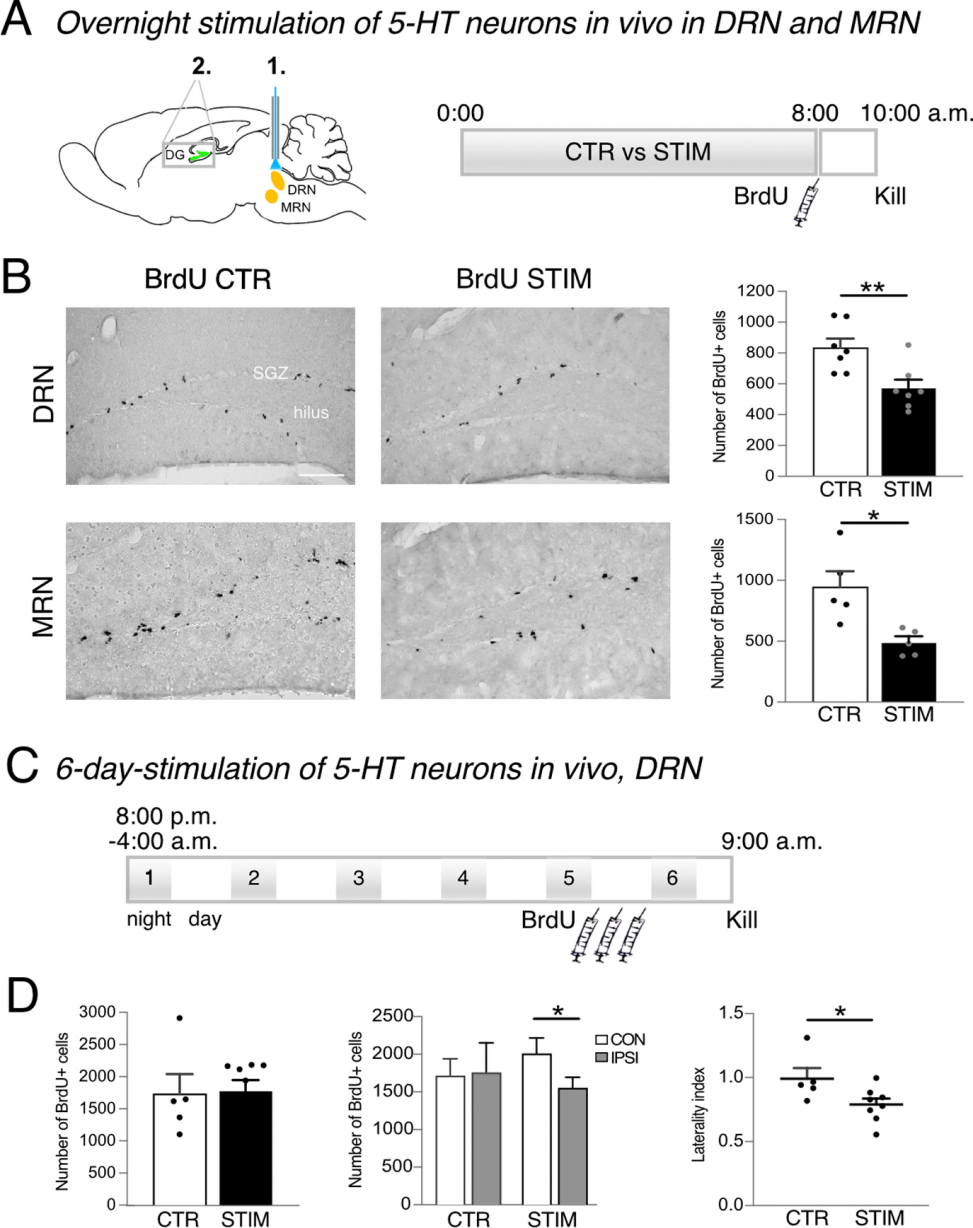
Stimulation of Tph2-ChR2-YFP neurons in the dorsal and median raphe nuclei (1, DRN, MRN) to examine the effects on cell proliferation in the dentate gyrus (2, DG). (A) Schematic drawing illustrating the target areas (1 and 2) and the experimental plan for overnight stimulation of 5-HT neurons. Light pulses of 20 Hz for 15 ms were transmitted for 30 s every 5 min for 8 h throughout the night. At 8 AM, mice received one intraperitoneal injection of BrdU to assess cell proliferation 2 h later. (B) Peroxidase staining to characterize BrdU-labeled cells in the subgranular zone (SGZ) of the hippocampus. Overnight light pulses to either the DRN or MRN significantly decreased the number of proliferating cells in the DG compared to the nonstimulated CTR groups. Scale bar represents 100 μm. BrdU, bromodeoxyuridine. Student’s *t*-tests, (*) *p* < 0.05, (**) *p* < 0.01. (C) In a separate set up, Tph2-ChR2-YFP neurons in the DRN were stimulated for 8 h over six consecutive nights. BrdU was injected three times on day 5, and brains were collected 24 h after the first injection. (D) No difference in cell proliferation in the hippocampus was detected between the control (CTR) and light-stimulated (STIM) groups. However, a laterality in the number of BrdU-positive cells per DG was observed following a six-night stimulation. Student’s *t*-tests, (*) *p* < 0.05. CON, contralateral; IPSI, ipsilateral.

In another in vivo study, we aimed to investigate whether activation of 5-HT neurons in freely moving Tph2-ChR2-YFP mice directly affects cell proliferation in the dentate gyrus (Figure 4A). Based on the above data, we determined that the most effective, reliable activation of 5-HT neurons is at 20 Hz with a pulse width of 15 ms. We conducted light stimulation of 5-HT neurons in mice for two time periods: (i) overnight and (ii) for six consecutive nights. Remarkably, overnight stimulation of 5-HT neurons in either DRN or MRN significantly decreased the number of BrdU-labeled cells in the hippocampus (DRN: CTR vs STIM, 834 ± 59 cells vs 573 ± 55 cells, *p* = 0.0070, *n* = 14; MRN: CTR vs STIM, 945 ± 130 cells vs 487 ± 48 cells, *p* = 0.0109, *n* = 10; Figure 4B). These results were somewhat unexpected, given the numerous studies proposing a neuromodulator role of 5-HT, as a result of its vast availability. Nonetheless, the data compare with our earlier study, where acutely lowered brain 5-HT led to a transient increase in the number of newborn cells in the dentate gyrus.^22^ In the current study, continuous stimulation of 5-HT neurons overnight might have led to either (i) constant 5-HT release at fiber projections to interneurons in the hilus^7^ subsequently inhibiting precursor cells in the dentate gyrus, (ii) varying the release of other neurotransmitters,^23,24^ or (iii) constant autoinhibition of 5-HT1A receptors on cell bodies, which ultimately shifts the balance between 5-HT release and inhibition, resulting in a lower firing rate of 5-HT neurons.^25^

### No Effects on Cell Proliferation Following One Week of Stimulation of Tph2-ChR2-YFP Neurons

When 5-HT neurons in the DRN were stimulated for six consecutive nights (Figure 4C)—building on our earlier study on running-induced cell proliferation that is 5-HT-dependent^13^—no difference in cell proliferation in the hippocampus was detected between the CTR and STIM group (1731 ± 312 cells vs 1776 ± 170 cells, *p* = 0.8926, *n* = 13; Figure 4D). However, a laterality in the number of BrdU-positive cells was observed following the continuous activation (laterality index CTR 1 ± 0.08 vs STIM 0.79 ± 0.04, *p* = 0.0440; Figure 4D), indicating fewer proliferating cells in the dentate gyrus ipsilateral to the optic fiber insertion (Figure 4D).

Topographically, 5-HT projections from the DRN exhibit more laterality compared to those from the MRN.^26,27^ Specifically, dorsal subnuclei have more unilateral projections to the forebrain than those located along the midline of the raphe nucleus.^27^ In our study, although the optic fiber ferrule was targeted at the center of either the DRN or MRN, it was practically inserted with an angle from the right hemisphere to prevent bleeding from the superior sagittal sinus vein. Given that light intensity weakens inside the brain, it is plausible that the right side of the DRN received more intense illumination than the left side. Acute overnight stimulation reduced the number of BrdU-positive cells in the entire dentate gyrus. However, after repeated stimulation over six consecutive nights, adaptive changes may have occurred, leading to inhibitory effects primarily in the ipsilateral hippocampus, which receives stronger projections. The observed laterality in our study could be the result of the interplay between adaptive changes and the topographical distribution of projections.

In this study, we utilized Tph2-ChR2-YFP mice to selectively activate 5-HT neurons, determining and establishing the frequency and duration of light pulses required for effective stimulation. Our findings reveal acute network dynamics between 5-HT raphe neurons and the hippocampus. Overnight stimulation directly affects cell proliferation in the dentate gyrus, resulting in a decrease in BrdU-positive cells. However, this decrease is absent with long-term light-activation of 5-HT neurons. These results relate to our earlier study, where acute reduction in brain 5-HT levels transiently increased the newborn cell number in the dentate gyrus, leading to enduring neuroanatomical and behavioral consequences.^22^ Defining downstream regulatory networks in vivo is crucial for understanding the effect of drugs like selective serotonin reuptake inhibitors (SSRIs), which initially exacerbate depressive symptoms before providing relief in 2–3 weeks. It remains to be seen whether a longer stimulation paradigm, corresponding to the typical latency in the therapeutic effects of SSRIs, could reverse the inhibitory effects of acute stimulation on cell proliferation and instead become a pro-proliferative stimulus for adult neurogenesis in rodents.

## Methods

### Animals and Housing Conditions

The BAC transgenic mouse line expressing the mhChR2::eYFP fusion protein directed to 5-HT neurons by the mouse *Tph2* promoter was obtained from Jackson Laboratory (https://www.jax.org/strain/014555). Young-adult Tph2-ChR2-YFP mice were randomly distributed throughout experiments (age 6–8 weeks, total *n* = 46). Four to six mice were housed in individually ventilated cages under laboratory conditions with a light/dark cycle of 12 h and free access to food and water. Consistent with requirements for animal care, cages were equipped with reusable polycarbonate enrichment products. All experiments were conducted in compliance with requirements set out in the European Communities Council Directive 2010/63/UE and were approved by the local animal welfare and ethical review body (Landesamt für Gesundheit and Soziales, LAGeSo, Berlin, Germany, No. G0300/13).

### Acute Brain Slices, Preparation, and Electrophysiology

Acute brain slices from *n* = 5 young-adult Tph2-ChR2-YFP mice were prepared to compare the firing patterns of DRN 5-HT vs non-5-HT neurons using patch clamp techniques. Mice were deeply anesthetized with isofluorane and decapitated, brains were removed and transferred into a chamber filled with ice-cold cutting solution (sucrose-based artificial cerebrospinal fluid, ACSF; in mM: 230 sucrose, 2.5 KCl, 1.25 NaH_2_PO_4_, 26 NaHCO_3_, 10 MgSO_4_, 0.5 CaCl_2_, 11 d-glucose, balanced with 95% O_2_–5% CO_2_; pH 7.4). The brainstems were sliced into 200-μm-thick coronal sections using a Vibratome (Model VF-310–0Z, Precision Instruments, Greenville, NC, USA). The brain slices were immediately transferred into a multiwell chamber and incubated in oxygenated (95% O_2_–5% CO_2_) ACSF (sucrose was replaced by NaCl; containing (in mM) 124 NaCl, 2.75 KCl, 1.25 NaH_2_PO_4_, 26 NaHCO_3_, 1.3 MgSO_4_, 2.0 CaCl_2_, 11 d-glucose). After 30–60 min in ACSF at 33 °C, and 1 h at room temperature, the slices were transferred with a pipet to a recording chamber installed on the stage of an upright microscope (Model SliceScope Pro 6000, Scientifica, Uckfield, U.K.).

YFP-positive cells located in the brainstem DRN were identified using a green-yellow-red LED pE-300ultra illumination system (Cool LED, Andover, U.K.) at an excitation of 570 nm generated by a YFP filter set (Scientifica). Patch pipettes were pulled from borosilicate glass capillaries (inner and outer diameter are 1.5 and 1.17 mm; Harvard Apparatus, Holliston, MA, USA) using the Smart Pull microelectrode puller (UniPix, Préverenges, Switzerland). The pipet solution contained the following concentration in mM: 125 K-gluconate, 5 NaCl, 2 MgCl_2_, 10 EGTA, 10 HEPES, 2 Na_2_ATP; adjusted to pH 7.2. The open resistance of the patch pipettes ranged from 3–5 MΩ. Whole-cell patch-clamp recordings were performed using a MultiClamp 700B amplifier connected to a Digidata 1550B digitizer (Molecular Devices, San Jose, CA, USA). Data sampled at 10 kHz and filtered at 4 kHz were analyzed using Clampfit 11.2 software (Molecular Devices, USA) in combination with NeuroExplorer v5 software (Nex Technologies, Colorado Springs, CO, USA).

Voltage- and current-clamp recordings of DRN neurons were obtained following blue light (470 nm) delivery through an optic fiber made of silica (400 μm diameter, 0.39 NA, N118L03; Thorlabs GmbH, Bergkirchen, Germany), positioned ∼1 mm above the brain slices. The light was either pulsed at frequencies ranging from 1 to 20 Hz for durations of 1 to 15 ms or continuously delivered for 1 s to induce inward currents at −65 mV or to elicit action potentials in the current-clamp mode (irradiance at 322 mW/mm^2^; LED light source BLS-FCS-0470–200 and BioLED Light Source Control Module BLS-13000–1E; Mightex, Toronto, Canada).

### Optetrode Preparation and Stimulation of Neurons in Anesthetized Mice

For in vivo analysis of 5-HT neurons, Tph2-ChR2-YFP-expressing cells in the DRN of anesthetized mice were light-stimulated, and extracellular field potentials were subsequently recorded via an optetrode. The optetrode enables simultaneous recording of neuronal electrical activity using a 4-channel tungsten tetrode fiber while delivering precise light stimulation to the same neurons using the optical fiber. An optic fiber (200 μm diameter and 0.66 numerical aperture [NA]; Prizmatix, Holon, Israel) was inserted into a 1.25 mm stainless steel fiber optic ferrule (Model SFLC230-10; Thorlabs GmbH, Bergkirchen, Germany) and placed into an electronic interface board (LabMaker GmbH, Berlin, Germany). To allow multichannel electrical recordings, 2 × 4 12.7 μm thin polyimide-coated tungsten microwires were assembled into two tetrodes (California Fine Wire, Grover Beach, CA, USA). The tetrodes were attached with gold pins to the electronic interface board (LabMaker GmbH), and colloidal gold (Neuralynx, Bozeman, MT, USA) was added onto microwires tips to lower the impedance to 200–250 kΩ. The two tetrodes were then fixed to the optic fiber with cyanoacrylate glue for in vivo optogenetic stimulation and recording.

Tph2-ChR2-YFP mice (*n* = 4) were anesthetized with an intraperitoneal injection (i.p.) of urethane (1.5 g/kg body weight; Sigma–Aldrich, Darmstadt, Germany) and placed into a small animal stereotaxic frame on a warm-water circulating platform set at 37 °C (RWD, Shenzhen, China). A craniotomy was performed above the DRN, and the optetrode was inserted at the following coordinates (from bregma, in mm): A/P −4.6, M/L + 1.2 at an angle of 23°, D/V −2.88. The inserted optetrode was connected to the optic fiber patch cable (200 μm diameter, 0.66 NA; Plexon, Dallas, TX, USA). Light stimulation at 10 mW and 465 nm was delivered via a PlexBright Table-Top LED Module and PlexBright 4 channel optogenetic controller (Plexon). Simultaneously, neuronal activity in the DRN was recorded via a HST/16D Gen2 digital head stage in combination with the OmniPlex D Neural Data Acquisition System (Plexon).

### Electrophysiology of 5-HT Neurons In Vivo, and Data Analysis

The recorded data were filtered using a wide-band filter between 0.10 and 7500 Hz. Single-unit spikes were then extracted offline from wide-band samples at 300–6000 Hz using Offline Sorter v4.5.0 (Plexon). Neurons were clustered into units using the waveform crossing method, and channels with a signal-to-noise ratio of <4 were excluded. None of the analyzed units contained more than 0.5% of events with interspike intervals below the refractory period of 1.2 ms.^28^ Extracellularly recorded spike waveforms were inverted using Offline Sorter to reflect the actual change in the membrane potential. The sorted spikes data were analyzed and visualized using NeuroExplorer v5 (Nex Technologies) and GraphPad Prism (GraphPad Software, San Diego, CA, USA).

### Optogenetic Stimulation of Raphe Neurons in Freely Moving Mice

To examine the effects on cell proliferation in the dentate gyrus, 5-HT neurons were light-activated in freely moving female Tph2-ChR2-YFP mice for one night or for six consecutive nights (Figures 4A and 4C, respectively). Mice for overnight stimulation were randomly assigned to four groups (*n* = 24): DRN stimulation (STIM), DRN control (CTR, without light), MRN stimulation, and CTR. The one-week group comprised STIM vs CTR for DRN activation (*n* = 13). Mice were deeply anesthetized with an i.p. injection of ketamine and xylazine (100:10 mg/kg body weight), placed into a small animal stereotaxic frame, and a craniotomy was performed as described above. Following the implantation of the optic fiber into either the DRN (refer to coordinates above) or MRN (coordinates from bregma, in mm: AP: −4.60, ML: +2.0 with an angle of 25°, DV: −4.45), the optic fiber ferrule was fixed with dental cement onto the skull (Vertex Self-Curing; Vertex Dental, Soesterberg, The Netherlands). Upon surgery, mice were housed singly for 1 week to prevent damage to the implanted optic fiber ferrule by other mice. Animals in the CTR group underwent the same experimental procedure. To initiate optogenetics, the optic fiber ferrules was connected to an optic patch cable of 0.8 m length, allowing mice moving freely within the cages. The cable was connected to a 465 nm PlexBright Compact LED Module attached to a PlexBright motorized carrousel (Plexon). To minimize possible light leaks, the connection between the ferrule and the patch cable was covered by metal sleeves (Prizmatix).^16^ For the duration of 8 h per night, light pulses at 10 mW, 20 Hz, 15 ms were delivered for 30 s every 5 min.

### Immunohistochemistry and Quantification of Proliferating Cells in the Dentate Gyrus

Tph2-ChR2-YFP mice in the overnight group received a single i.p. injection of BrdU (5-bromo-2′-deoxyuridine, 50 mg/kg bodyweight; Sigma–Aldrich) following an 8-h stimulation and were perfused 2 h later. For the one-week stimulation group, BrdU was injected three times, 6 h apart on day 5, and mice were killed 24 h after the first injection. Mice were deeply anesthetized and perfused transcardially with 0.9% saline. Brains were removed and placed into 4% paraformaldehyde overnight (followed by 30% sucrose). BrdU immunohistochemistry was performed following the peroxidase method according to an established protocol.^13^ Briefly, one-in-six series of sequential 40 μm coronal brain sections were stained free-floating. DNA was denatured in 2 N HCl for 20 min at 37 °C, sections were rinsed in 0.1 M borate buffer, and washed in Tris-buffered saline (TBS). The primary antibody anti-BrdU (rat, 1:500; Bio-Rad AbD Serotec, Neuried, Germany) and biotinylated secondary antibody were diluted in TBS containing 3% donkey serum and 0.1% Triton X-100. Immunoreactive cells were counted throughout the rostro-caudal extent of the dentate gyrus. The total number of labeled cells was estimated by multiplying cell counts by six.

### Statistical Analysis

Student’s *t*-test was used for individual pairwise comparisons (GraphPad Prism software v9.0.2, San Diego, CA, USA). All values are expressed as mean ± SEM *p* values of ≤0.05 were considered statistically significant.

## Abbreviations

5-HT: 5-hydroxytryptamine (serotonin)
BrdU: bromodeoxyuridine
ChR2: channelrhodopsin-2
DRN: dorsal raphe nuclei
MRN: median raphe nuclei
TPH2: tryptophan hydroxylase 2
YFP: yellow fluorescent protein
